# Supplementary material for: Synthesis, Characterization and Biological Investigation of the Platinum(IV) Tolfenamato Prodrug–Resolving Cisplatin-Resistance in Ovarian Carcinoma Cell Lines
Source: Int J Mol Sci. 2023 Mar 16;24(6):5718. doi: 10.3390/ijms24065718 (PMC10056020; doi:10.3390/ijms24065718)
Supplement: Supplementary file 1 [file ijms-24-05718-s001.zip › ijms-2228211-supplementary.pdf]

Electronic Supplementary Information

Synthesis, characterization and biological investigation of the platinum(IV) tolfenamato prodrug – resolving cisplatin-resistance in ovarian carcinoma cell lines

Marie-Christin Barth [1,#], Norman Häfner [2,#], Ingo B. Runnebaum [2,*], and Wolfgang Weigand [1,*]

[1]Department of Inorganic and Analytical Chemistry, Friedrich Schiller University Jena, Humboldtstrasse 8, 07743 Jena, Germany
E-mail: wolfgang.weigand@uni-jena.de
Fax: +49 3641 948102
Tel: +49 3641 948160

[2]Department of Gynecology and Reproduction Medicine, Jena University Hospital, Am Klinikum 1, 07747 Jena, Germany
E-mail: Direktion-Gyn@med.uni-jena.de

# These authors contributed equally to this work.

Contents

Methods and materials 2
Synthetic procedures 3
^{1}H and $^{13}C\{^{1}H\}$ NMR spectra 4
Stability investigation of complex **7** in different buffer solutions 17
Reduction experiment of complex **7** with ascorbic acid (UHPLC-HRMS) 18
COX-1/2 inhibition 18
Cell death rate after cisplatin treatment 19
References 19

Methods and materials

EI mass spectra. Samples were measured on a MS system from Thermo (Bremen, Germany) consisting of an ITQ 900 ion trap mass spectrometer or an SSQ710 single quadrupole mass spectrometer using the direct insertion probe (DIP) technique. The sample was placed on the rhenium wire and evaporated using a current slope of 100 mA·sec^{-1} (from 0 to 800 mA); MS-parameters: scan range 50-900 *m/z* for ITQ and 20-500 *m/z* for SSQ710 respectively. Ion source was set to 200 °C.

ESI mass spectra. Automated samples were carried out by dissolving about 0.2 mg sample in 1 mL MeOH; 2 µL of the solvent were sampled by Agilent (Waldborn, Germany) 1100 G1313 A autosampler and delivered to mass spectrometer by binary pump module G1312A with isocratic elution (90 % MeOH, 10 % water with 0.1 % formic acid). Sample was eluted with 20 µL/min to Bruker (Bremen, Germany) MAXIS mass spectrometer. ESI_{pos}: 500 V end plate offset, 4500 V capillary voltage, 0.4 bar nebulizer pressure, 4 L/min dry gas flow, 180 °C dry temperature. Tune values for ion transfer were set to 200 Vpp Funnel 1 RF, 0.0 eV is CID Energy, 200 Vpp Multipole RF, eV Quadrupole Ion Energy with low mass setting of 100 *m/z*. Collision energy was set to 8 eV, 170 Vpp collision RF, 90 µs transfer time and 5 µs pre pule storage time. Mass window from 100 to 1500 *m/z* was monitored. Internal mass calibration was performed for each spectrum using sodium formate after sample elution with HPC-calibration algorithm using Bruker Compass Data Analysis 4.3 software.

LC-MS. Ultra-high performance liquid chromatography coupled with high resolution mass spectrometry was carried out using a THERMO (Bremen, Germany) UltiMate HPG-3400 RS binary pump, WPS-3000 auto sampler which was set to 10 °C and which was equipped with a 25 µL injection syringe and a 100 µL sample loop. The column was kept at 25 °C within the column compartment TCC-3200. Chromatography column was used THERMO Accucore® C-18 RP (100 × 2.1 mm; 2.6 µm) using the gradient in **Table S1** at a constant flow rate of 0.4 mL/min. Eluent A was water, with 2 % acetonitrile and 0.1 % formic acid. Eluent B was pure acetonitrile.

Mass spectra were recorded with THERMO QExactive plus orbitrap mass spectrometer coupled to a heated electrospray source (HESI). Column flow was switched at 0.5 min from waste to the MS and at 11.5 min again back to the waste, to prevent source contamination. For monitoring two full scan modes were selected with the following parameters. Polarity: positive; scan range: 100 to 1500 *m/z*; resolution: 70,000; AGC target: 3×10^6; maximum IT: 200 ms. General settings: sheath gas flow rate: 60; auxiliary gas flow rate 20; sweep gas flow rate: 5; spray voltage: 3.0 kV; capillary temperature: 360 °C; S-lens RF level: 50; auxiliary gas heater temperature: 400 °C; acquisition time frame: 0.5 - 11.5 min. For negative mode, all values were kept instead of the spray voltage which was set to 3.3 kV.

At the beginning and at the end a pooled (equal mixture (v/v) of all individual samples) sample was measured to guarantee system integrity. This pooled sample was additionally used to measure in data dependent mode MS^2 spectra with the following settings: scan range: auto, resolution: 17,500; AGC target: 3×10^6; maximum IT: 32 ms, loop count = 5, preferred charge state = 1, dynamic exclusion: 30 sec.

Table S1. Gradient for UHPLC / HRMS measurement.

Time / min	solvent B / %
0	0
0.2	0
8.0	100
11.0	100
11.1	0
12.0	0

Synthetic procedures

Preparation of O1 [1,2]

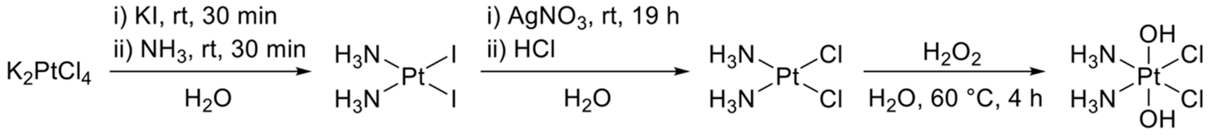

O1

K_2PtCl_4 (1 g, 2.4 mmol) and KI (2.4 g, 14.4 mmol) were stirred in water for 30 min at room temperature. After the addition of 2 M NH_3 (5 mL), the reaction mixture was stirred for another 30 min. The precipitate was filtered, washed with water, EtOH and ether (1.06 g, 92%).
[Pt(NH_3)I_2] (1.06 g, 2.2 mmol) and $AgNO_3$ (704 mg, 4.14 mmol) were stirred in water at room temperature overnight. After filtration, the filtrate was concentrated, and hydrochloric acid was added dropwise until precipitation of a yellow solid. The product was washed with water, EtOH and ether (500 mg, 76%).
Cisplatin (500 mg, 1.7 mmol) was oxidized by hydrogen peroxide (36 mL) in water at 60 °C for 4 h. The reaction mixture was stored at 4°C for precipitation of the product, which was washed with water, EtOH and ether (300 mg, 53%).

Preparation of compound O2 [3]

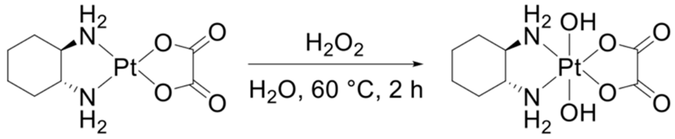

O2

Oxaliplatin (0.5 g, 1.25 mmol) was suspended in distilled water. After the addition of H_2O_2 (18 mL), the reaction mixture was stirred at 60 °C for 2 h. The solvent was evaporated to a volume of 2 mL and the product was precipitated by addition of EtOAc and washed with EtOH and ether (0.4 g, 74%).

^{1}H and ^{13}C{^{1}H} NMR spectra

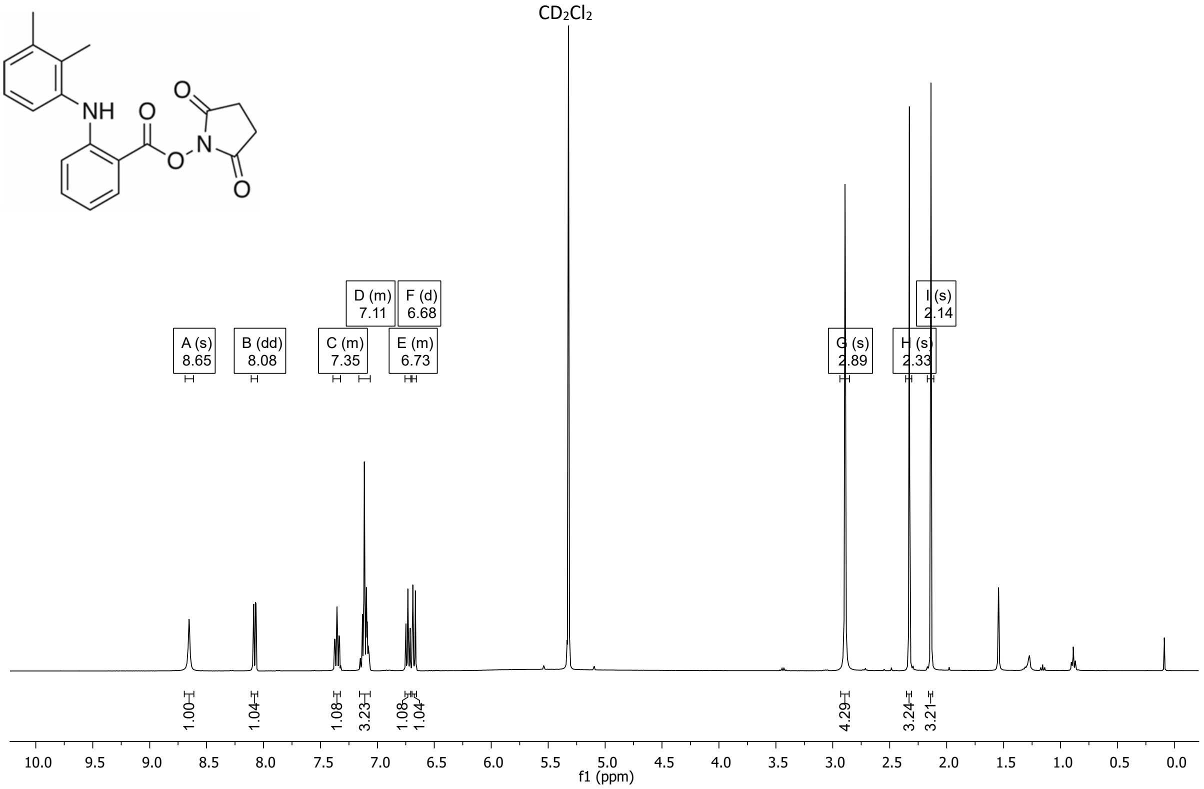

Figure S1. ^{1}H NMR spectrum of **1** (CD_2Cl_2, 400 MHz).

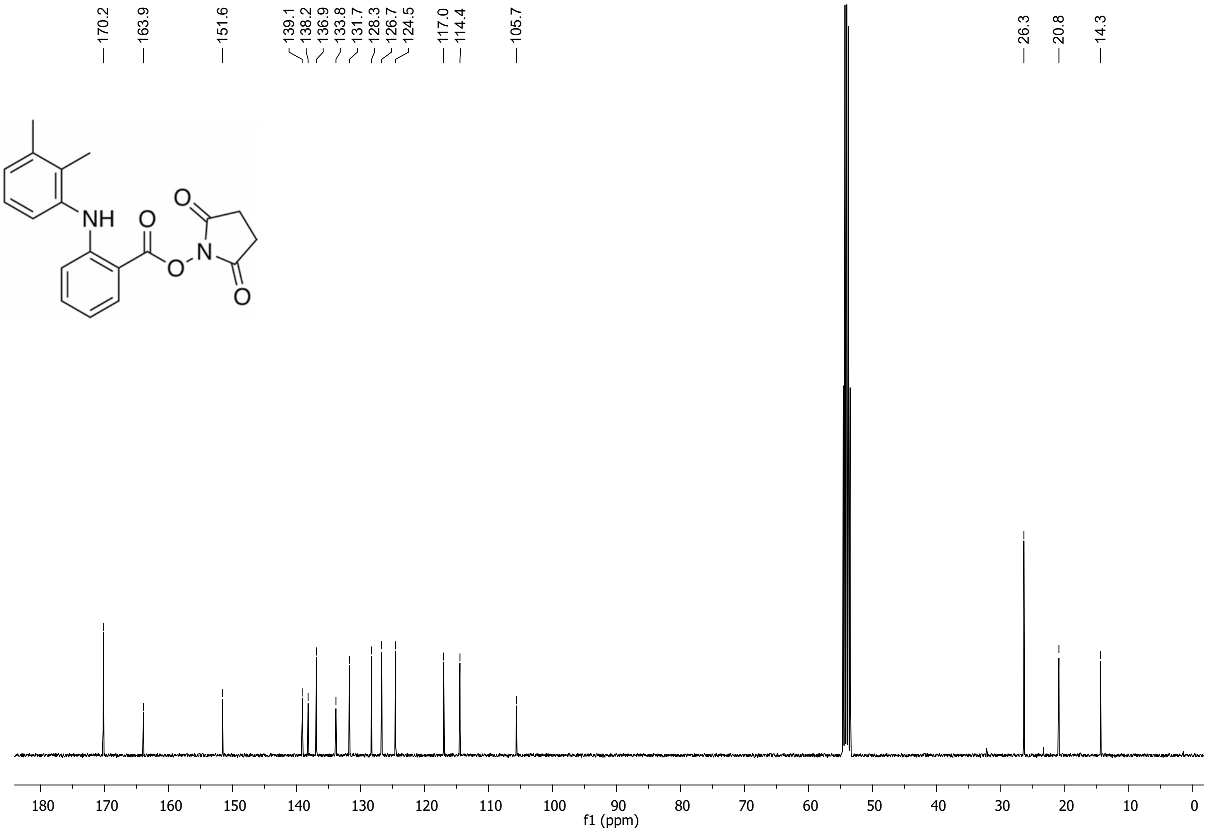

Figure S2. ^{13}C{^{1}H} NMR spectrum of **1** (CD_2Cl_2, 101 MHz).

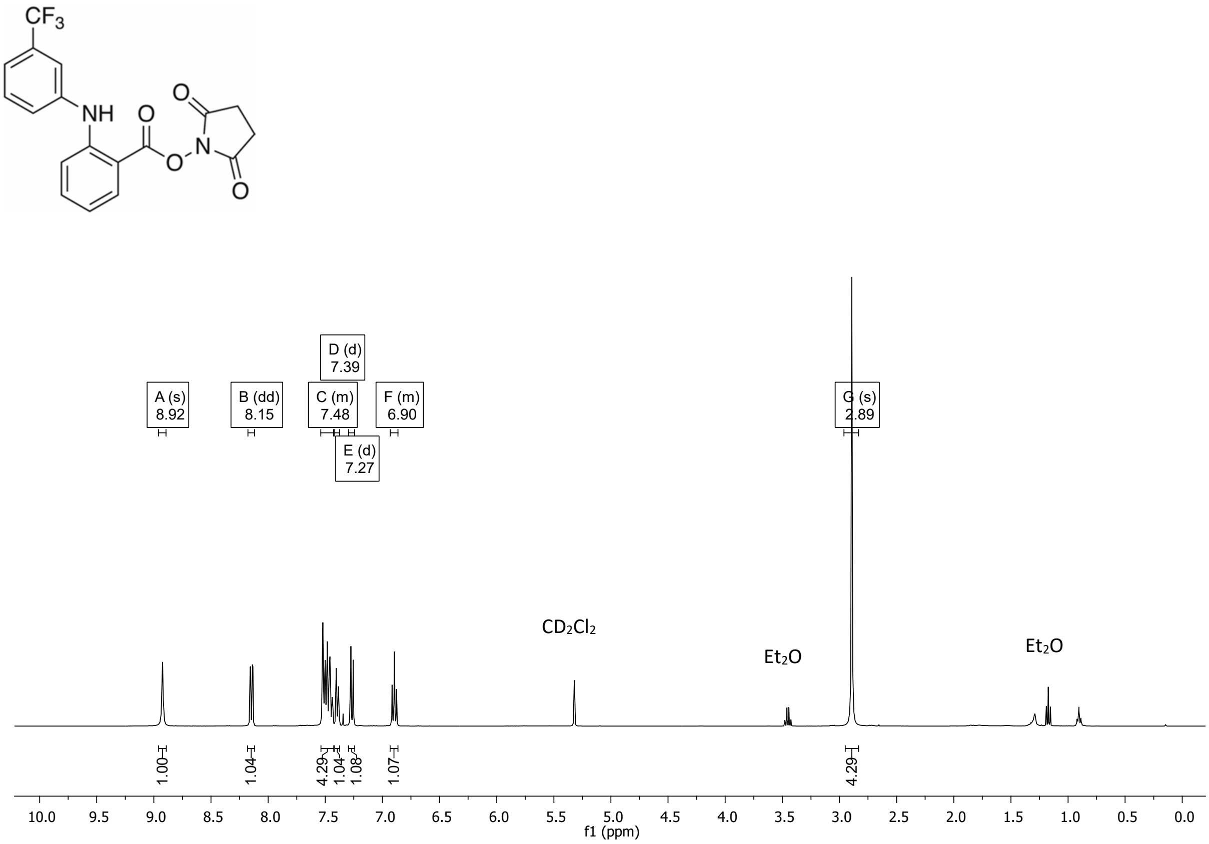

Figure S3. ^{1}H NMR spectrum of **2** (CD_2Cl_2, 400 MHz).

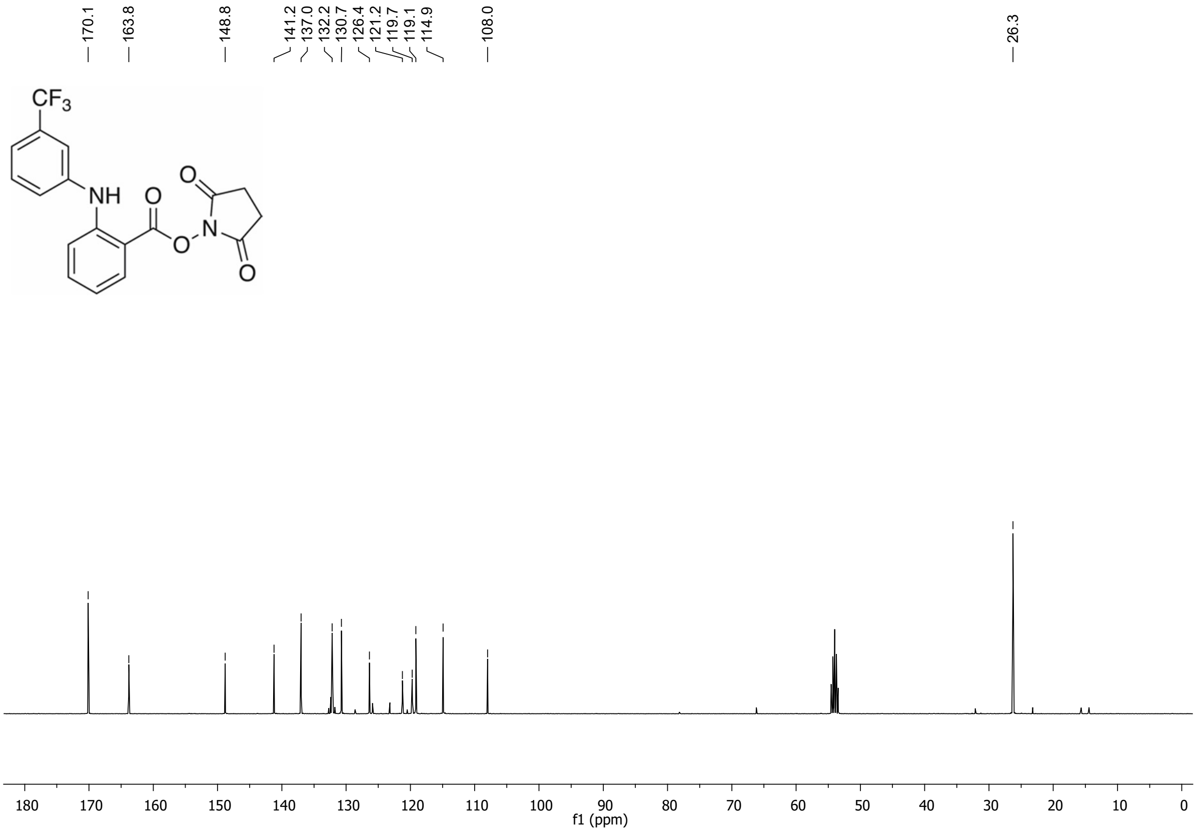

Figure S4. $^{13}C\{^{1}H\}$ NMR spectrum of **2** (CD_2Cl_2, 101 MHz).

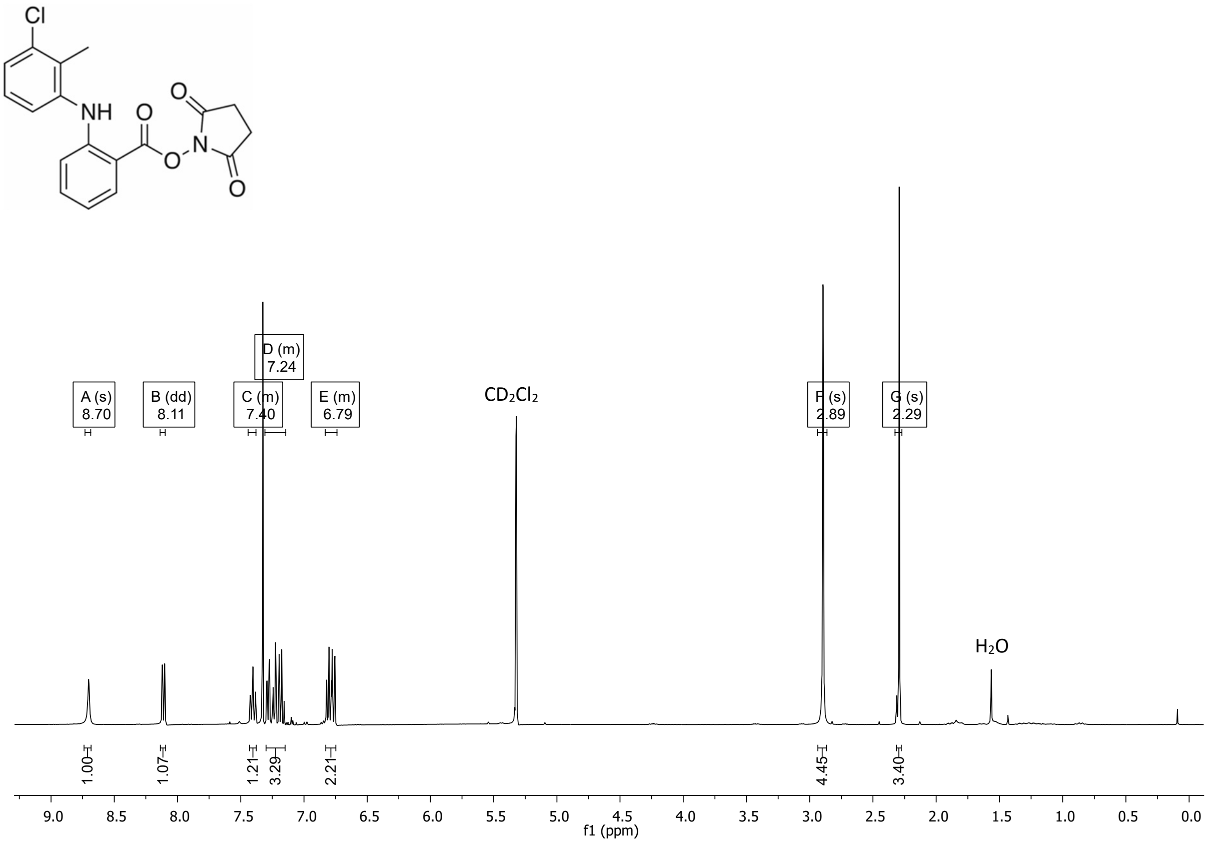

Figure S5. ^{1}H NMR spectrum of **3** (CD_2Cl_2, 400 MHz).

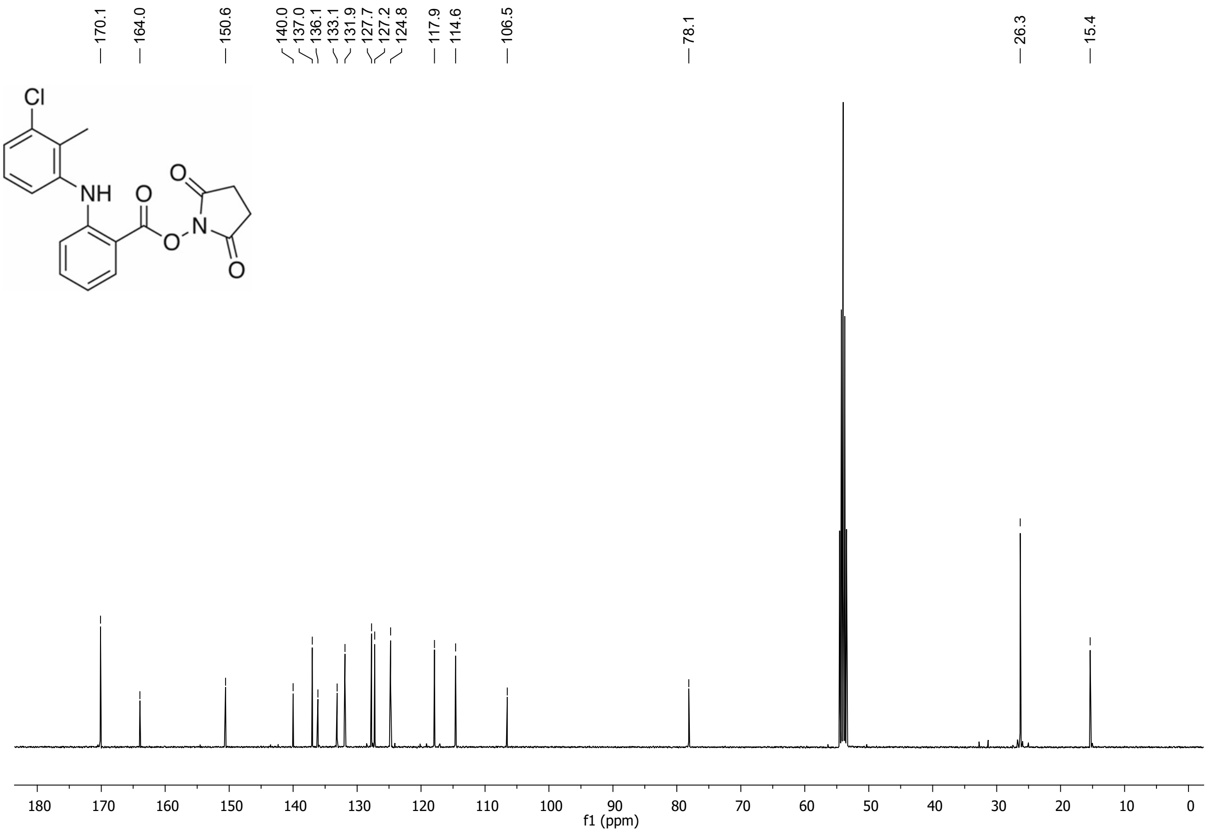

Figure S6. $^{13}C\{^{1}H\}$ NMR spectrum of **3** (CD_2Cl_2, 101 MHz).

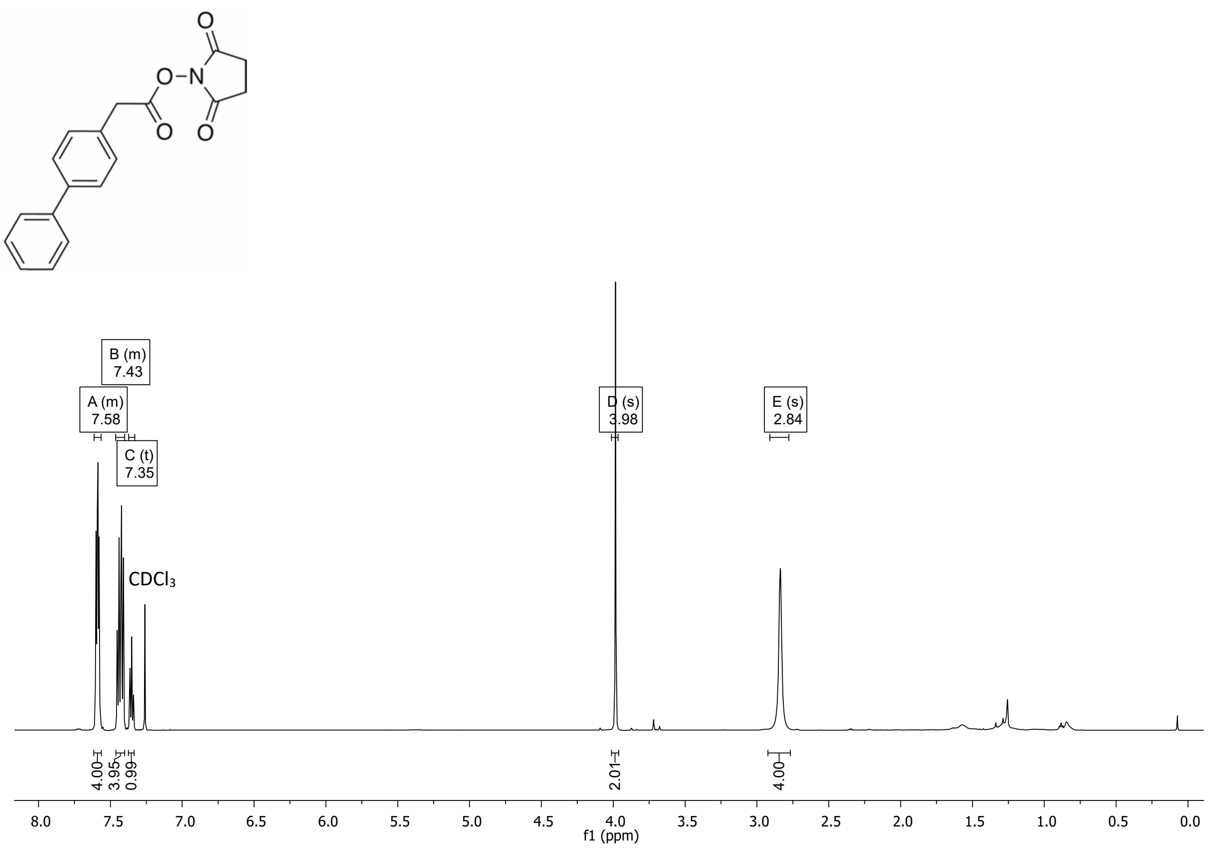

Figure S7. ^{1}H NMR spectrum of **4** ($CDCl_3$, 600 MHz).

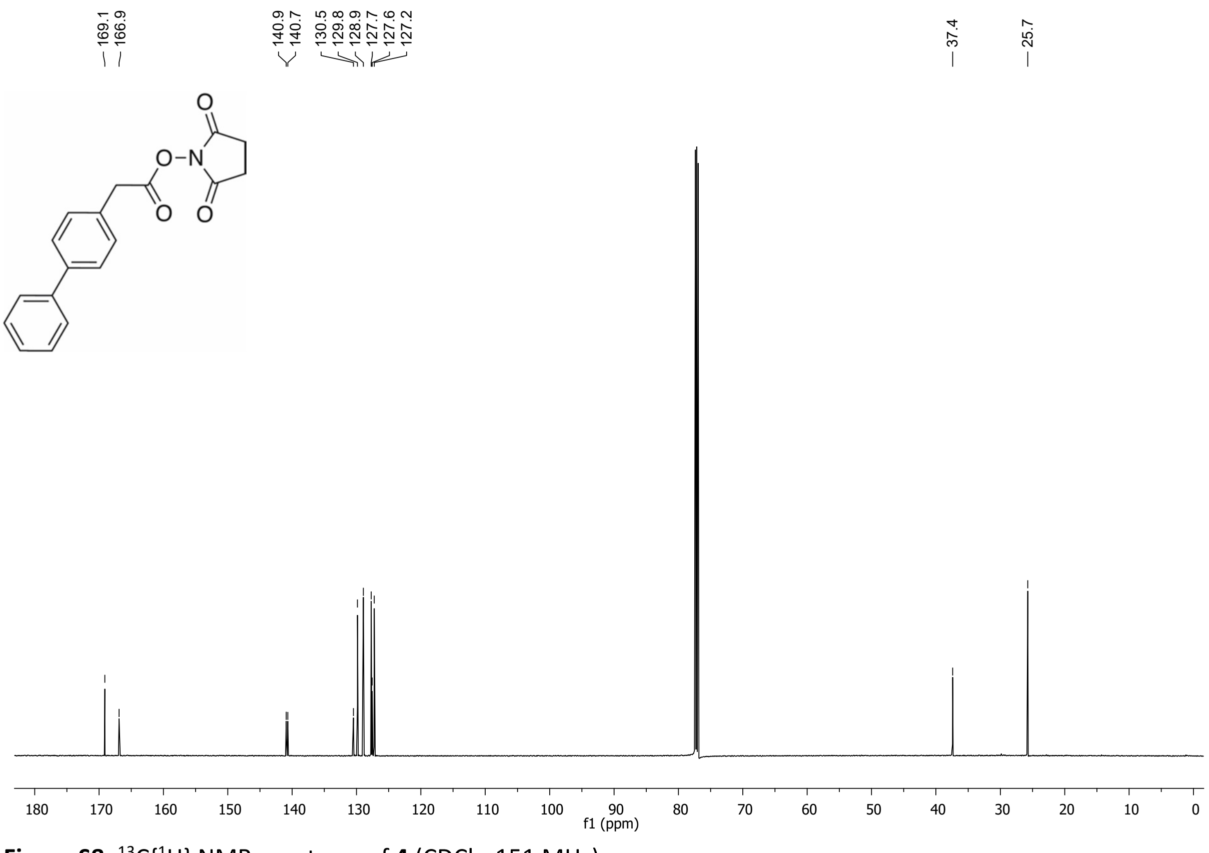

Figure S8. $^{13}C\{^{1}H\}$ NMR spectrum of **4** ($CDCl_3$, 151 MHz).

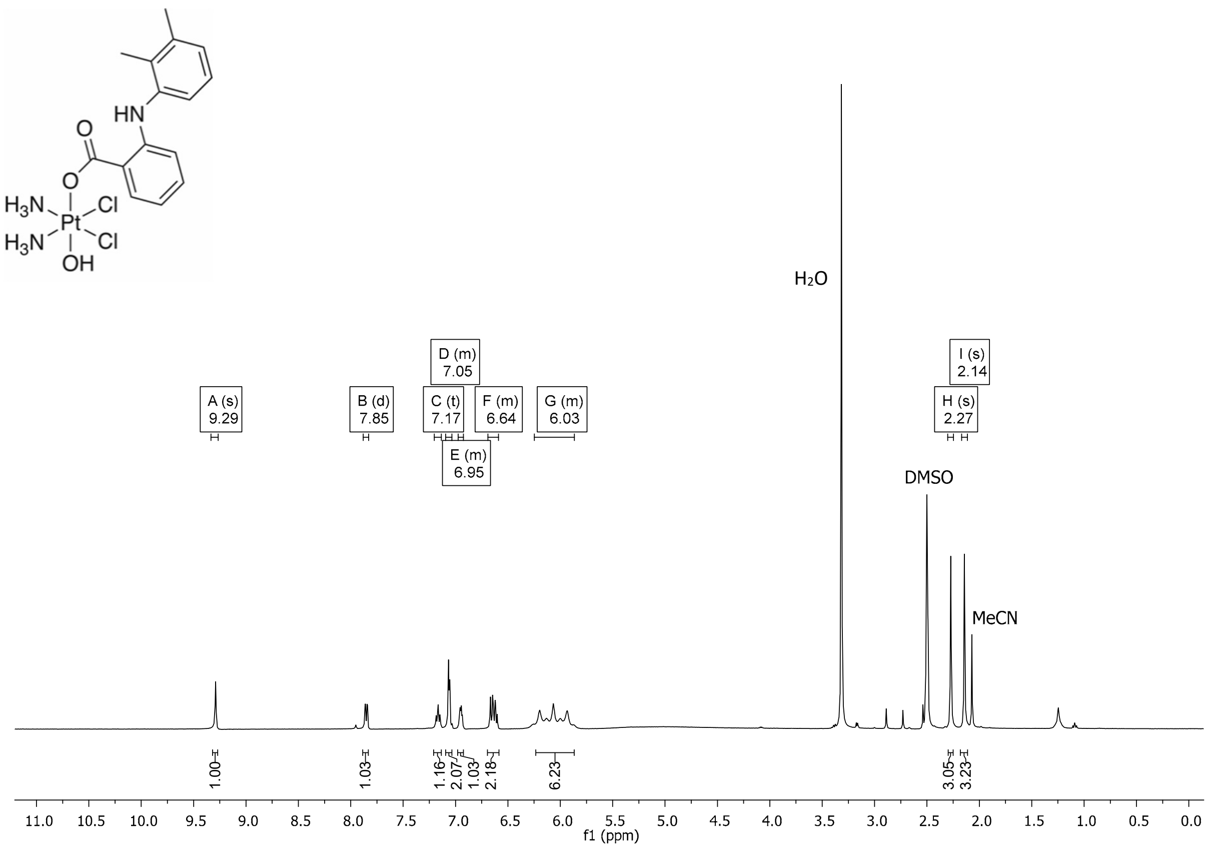

Figure S9. ^{1}H NMR spectrum of **5** (DMSO-d$_6$, 400 MHz).

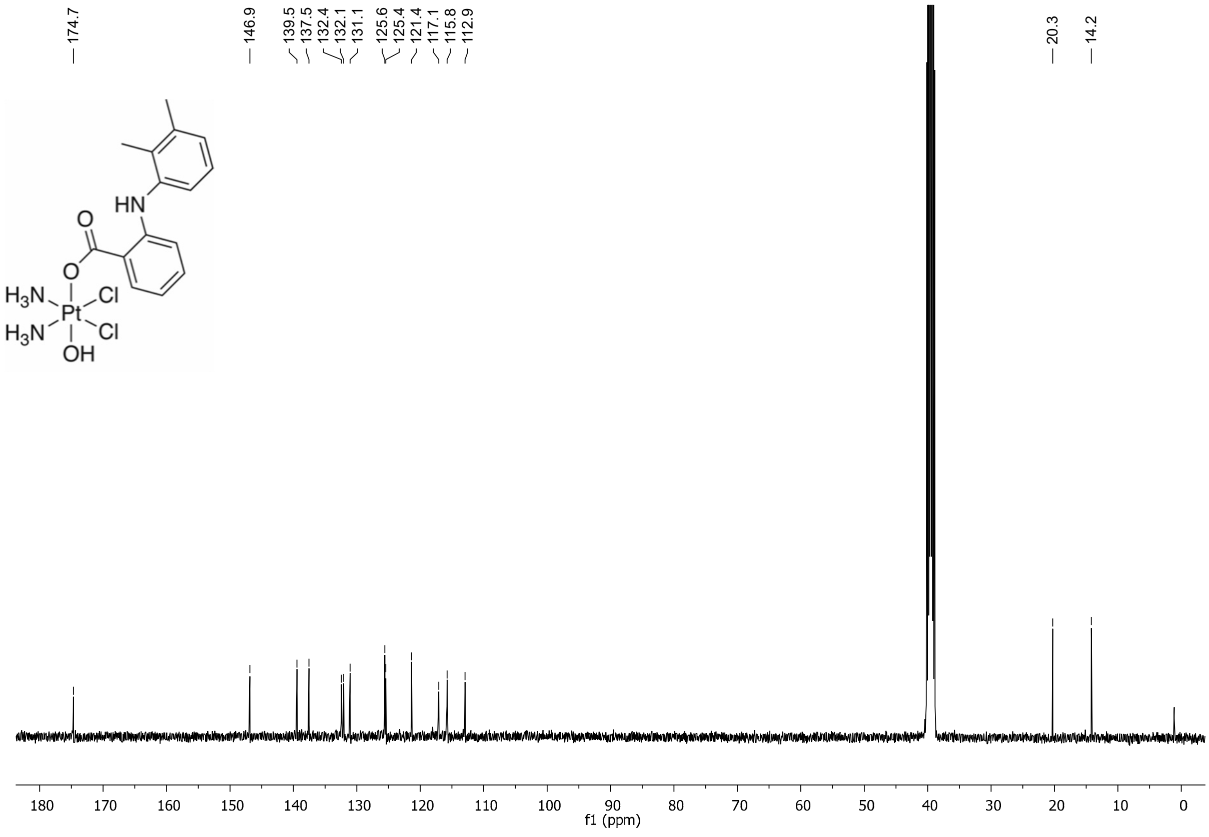

Figure S10. $^{13}C\{^{1}H\}$ NMR spectrum of **5** (DMSO-d$_6$, 101 MHz).

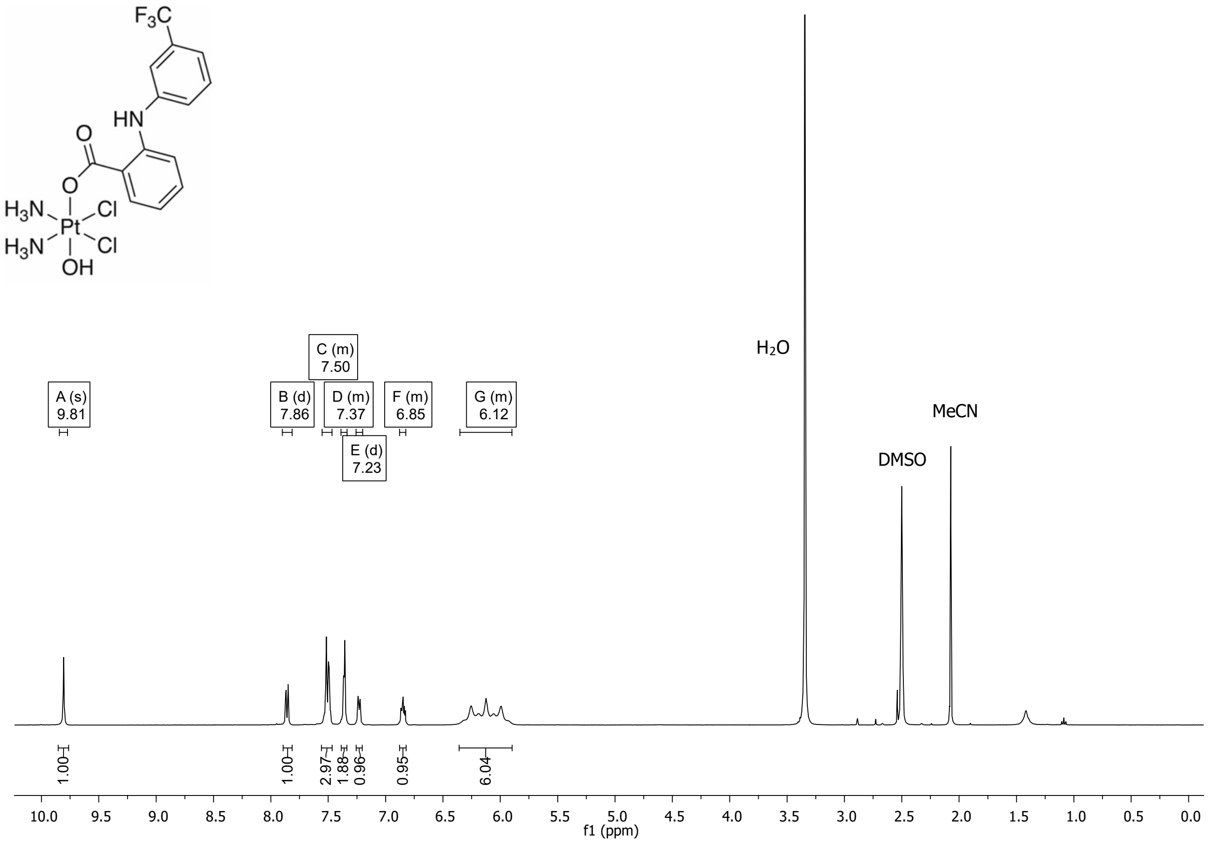

Figure S11. ^{1}H NMR spectrum of **6** (DMSO-d_6, 400 MHz).

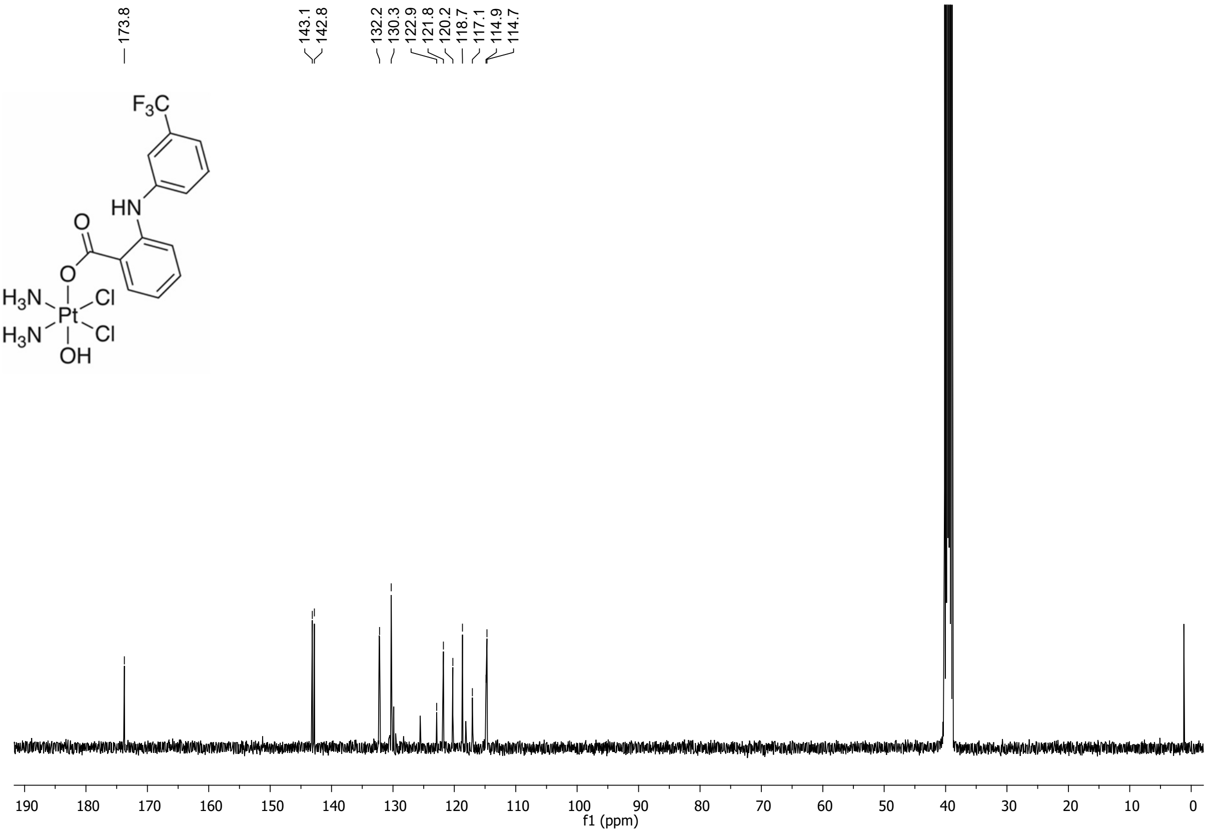

Figure S12. $^{13}C\{^{1}H\}$ NMR spectrum of **6** (DMSO-d_6, 101 MHz).

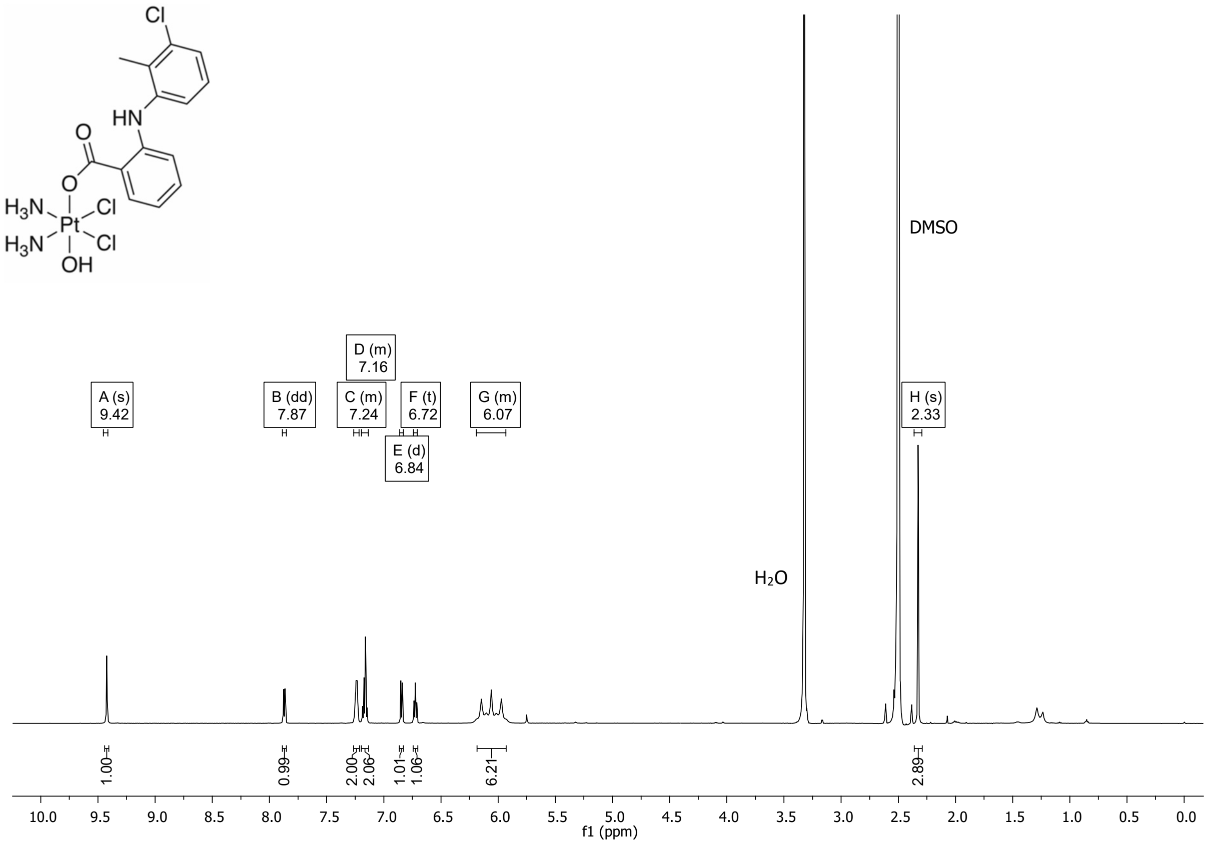

Figure S13. ^{1}H NMR spectrum of **7** (DMSO-d_6, 600 MHz).

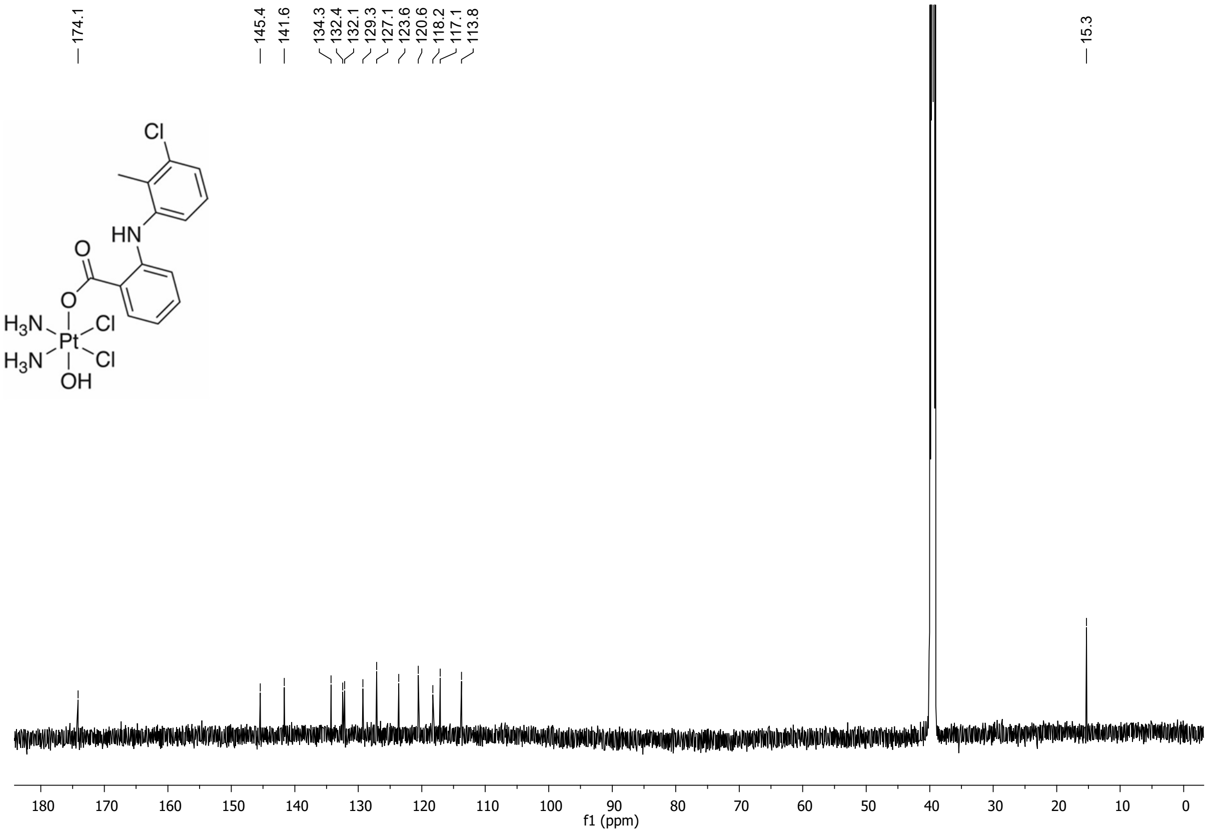

Figure S14. $^{13}C\{^{1}H\}$ NMR spectrum of **7** (DMSO-d_6, 151 MHz).

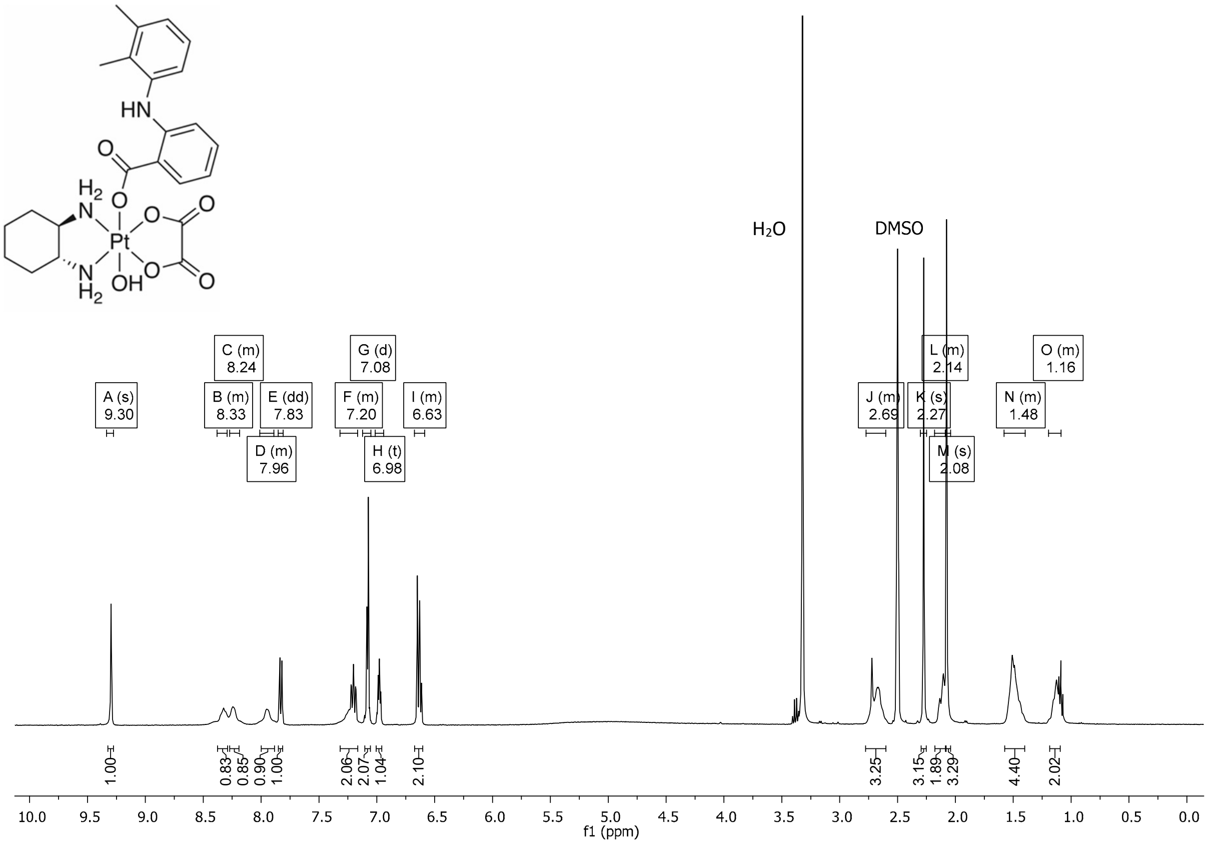

Figure S15. ^{1}H NMR spectrum of **8** (DMSO-d_6, 400 MHz).

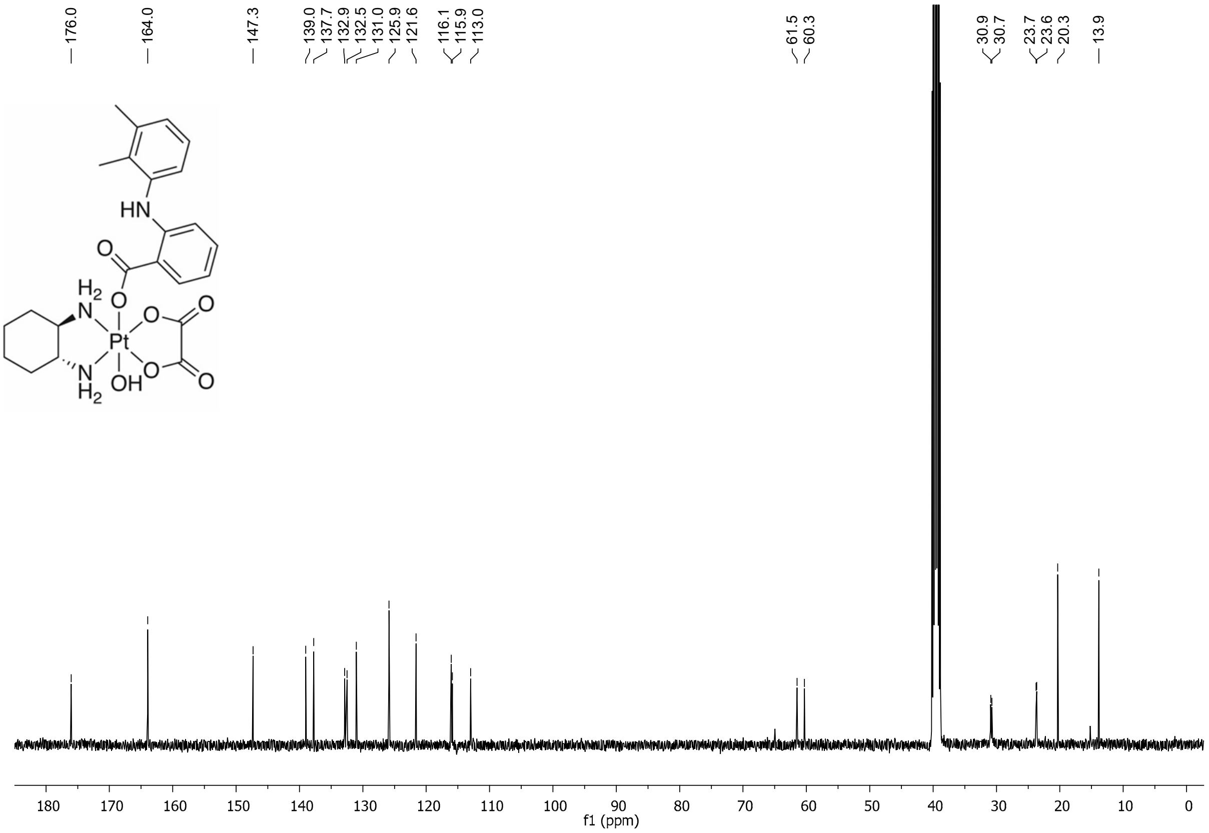

Figure S16. $^{13}C\{^{1}H\}$ NMR spectrum of **8** (DMSO-d_6, 101 MHz).

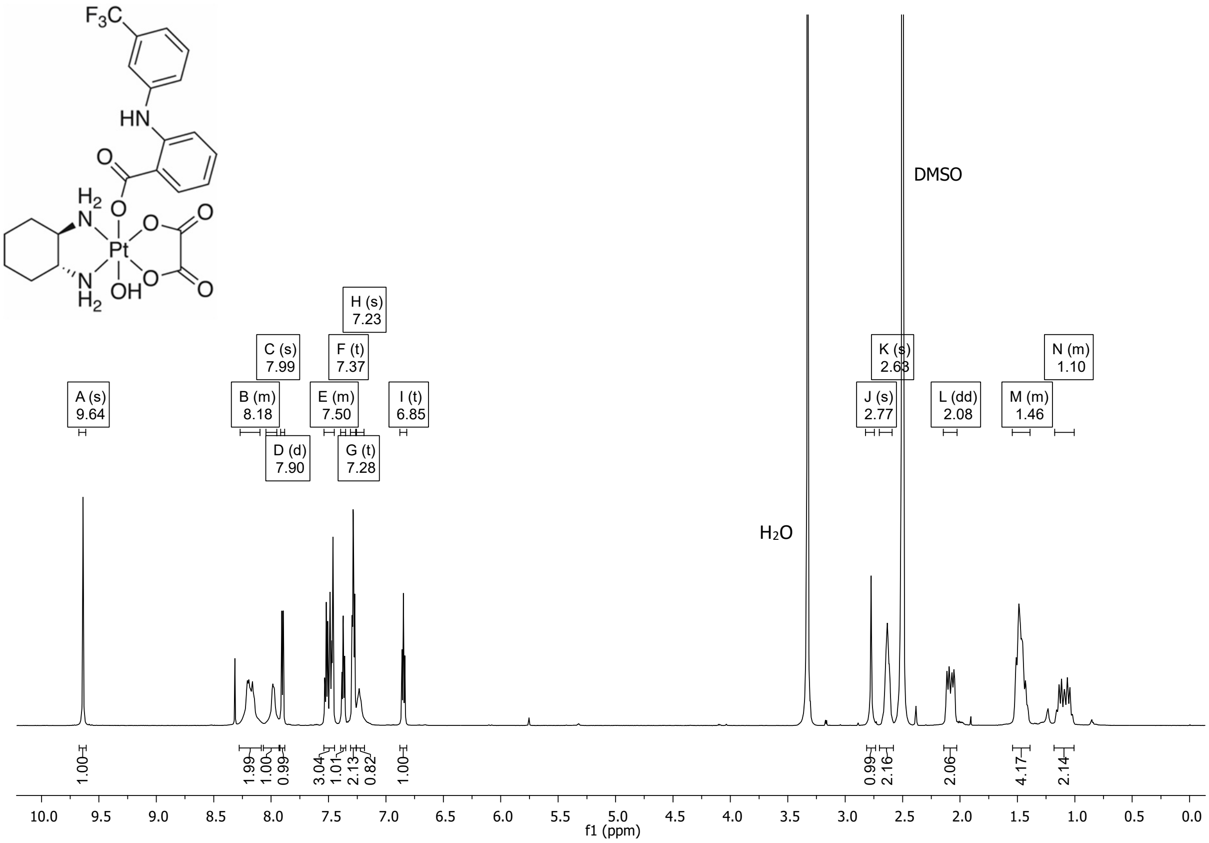

Figure S17. ^{1}H NMR spectrum of **9** (DMSO-d_6, 600 MHz).

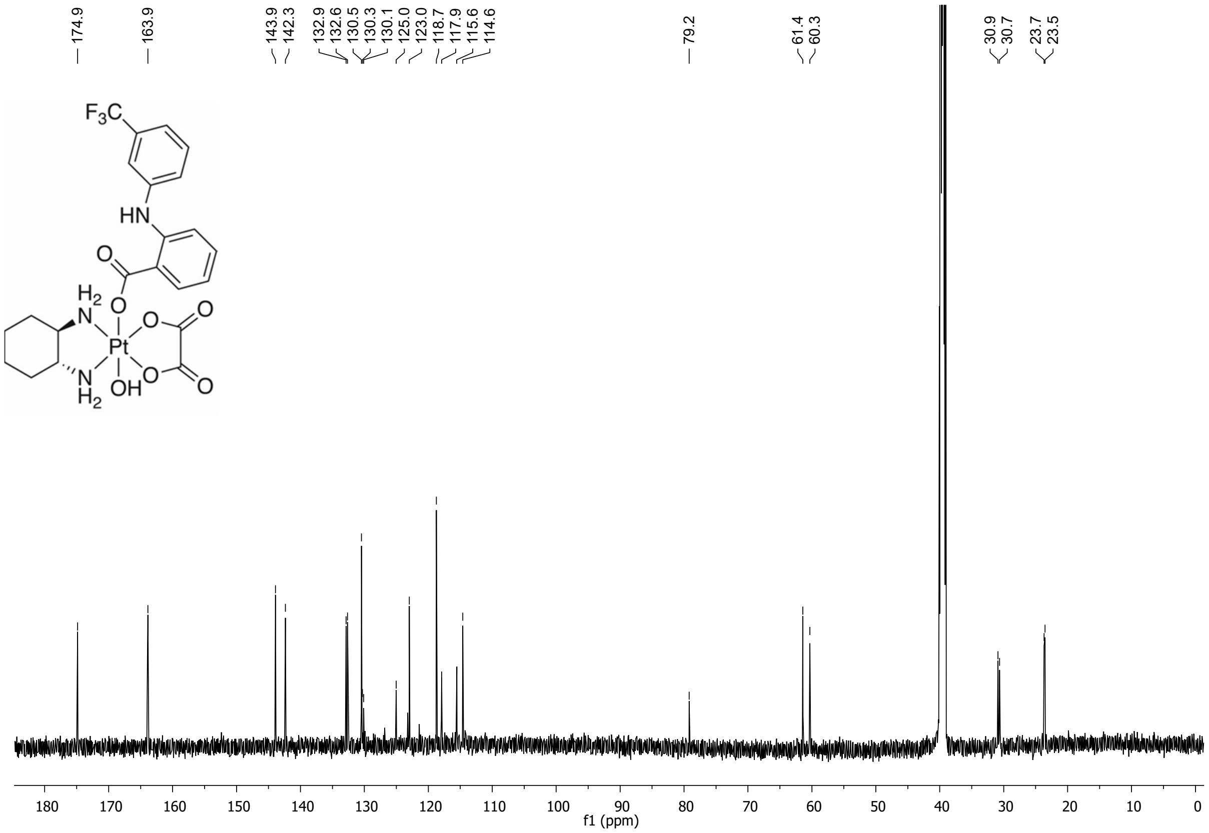

Figure S18. $^{13}C\{^{1}H\}$ NMR spectrum of **9** (DMSO-d_6, 151 MHz).

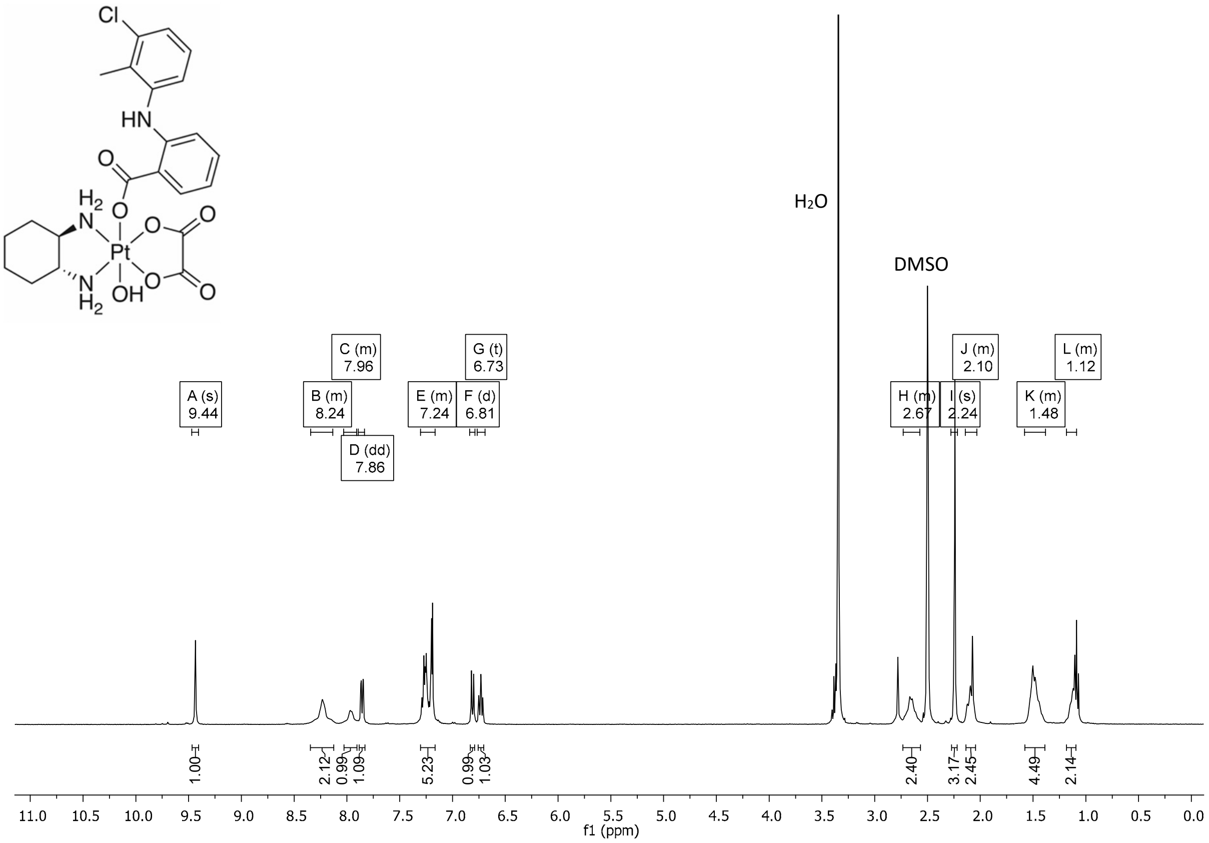

Figure S19. ^{1}H NMR spectrum of **10** (DMSO-d$_6$, 400 MHz).

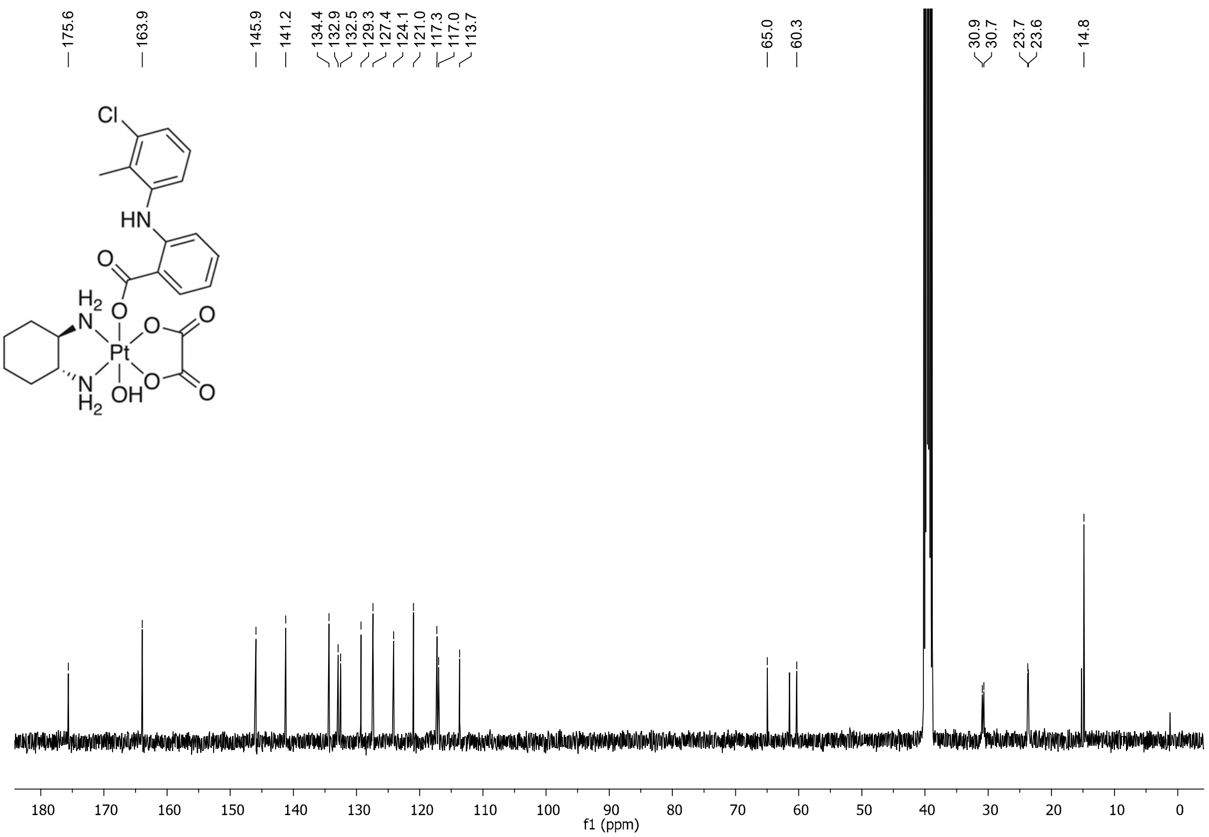

Figure S20. ^{13}C{^{1}H} NMR spectrum of **10** (DMSO-d$_6$, 101 MHz).

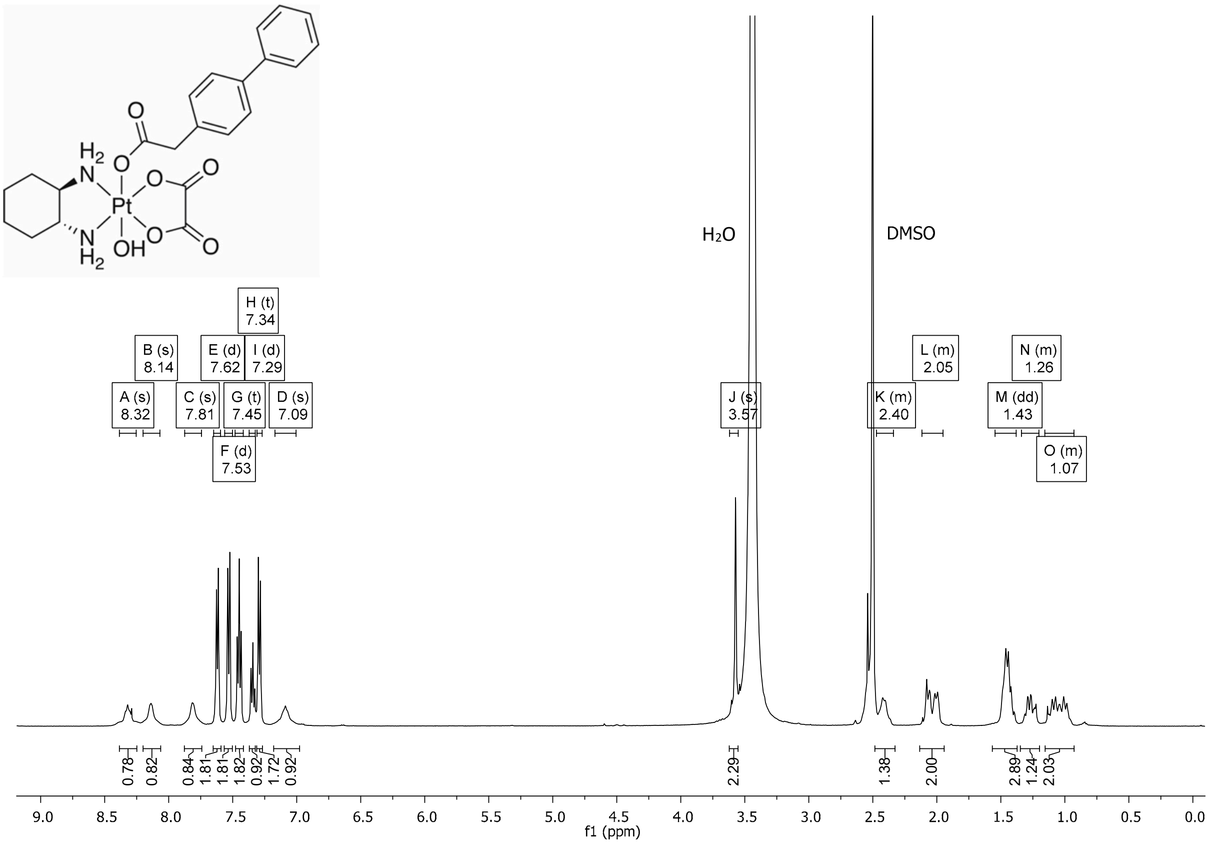

Figure S21. ^{1}H NMR spectrum of **11** (DMSO-d$_6$, 500 MHz).

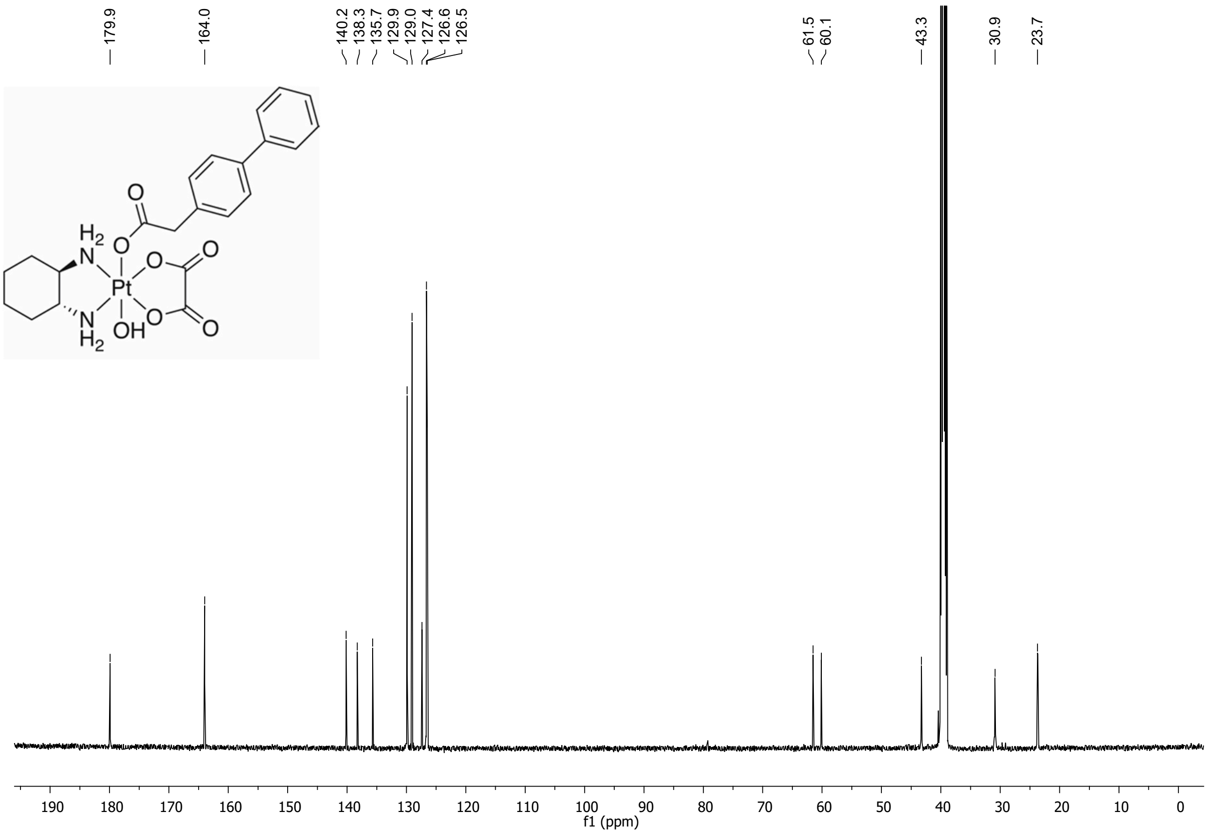

Figure S22. ^{13}C{^{1}H} NMR spectrum of **11** (DMSO-d$_6$, 125 MHz).

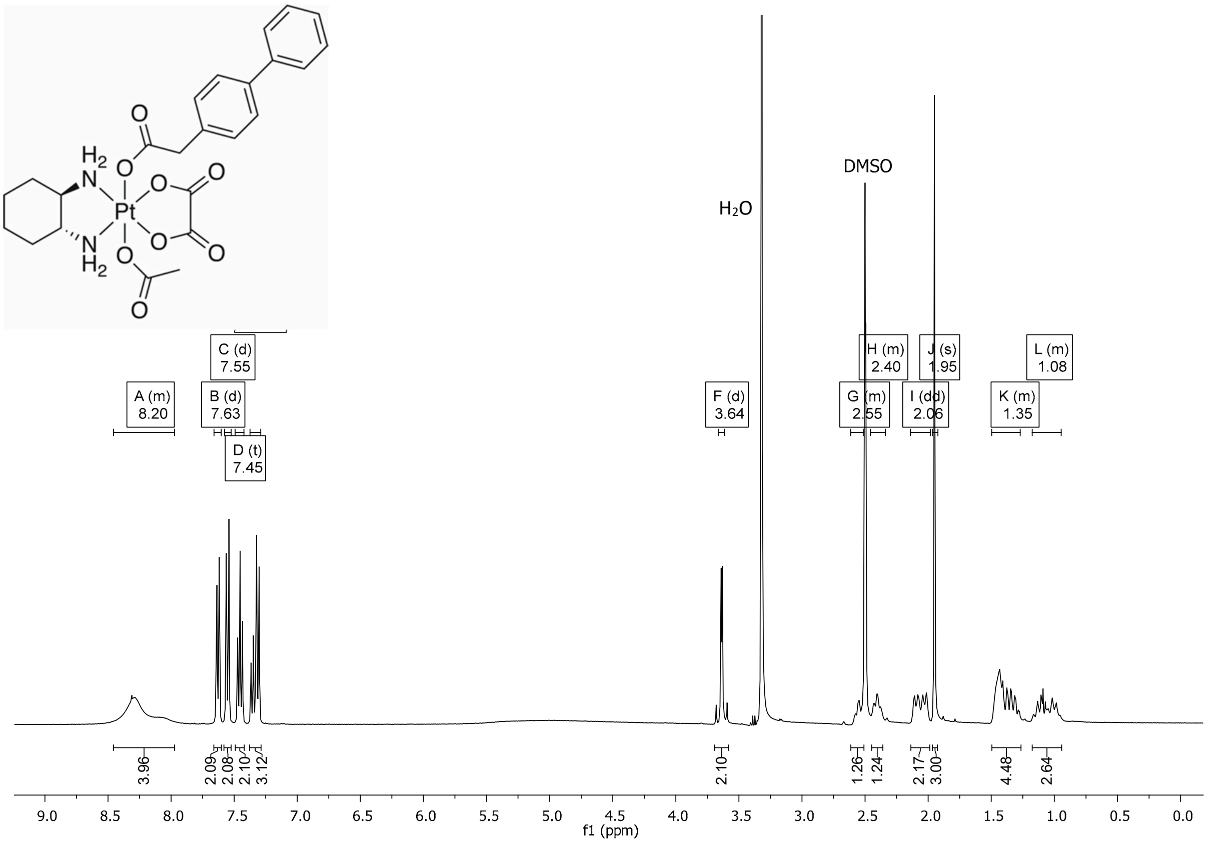

Figure S23. ^{1}H NMR spectrum of **12** (DMSO-d$_6$, 400 MHz).

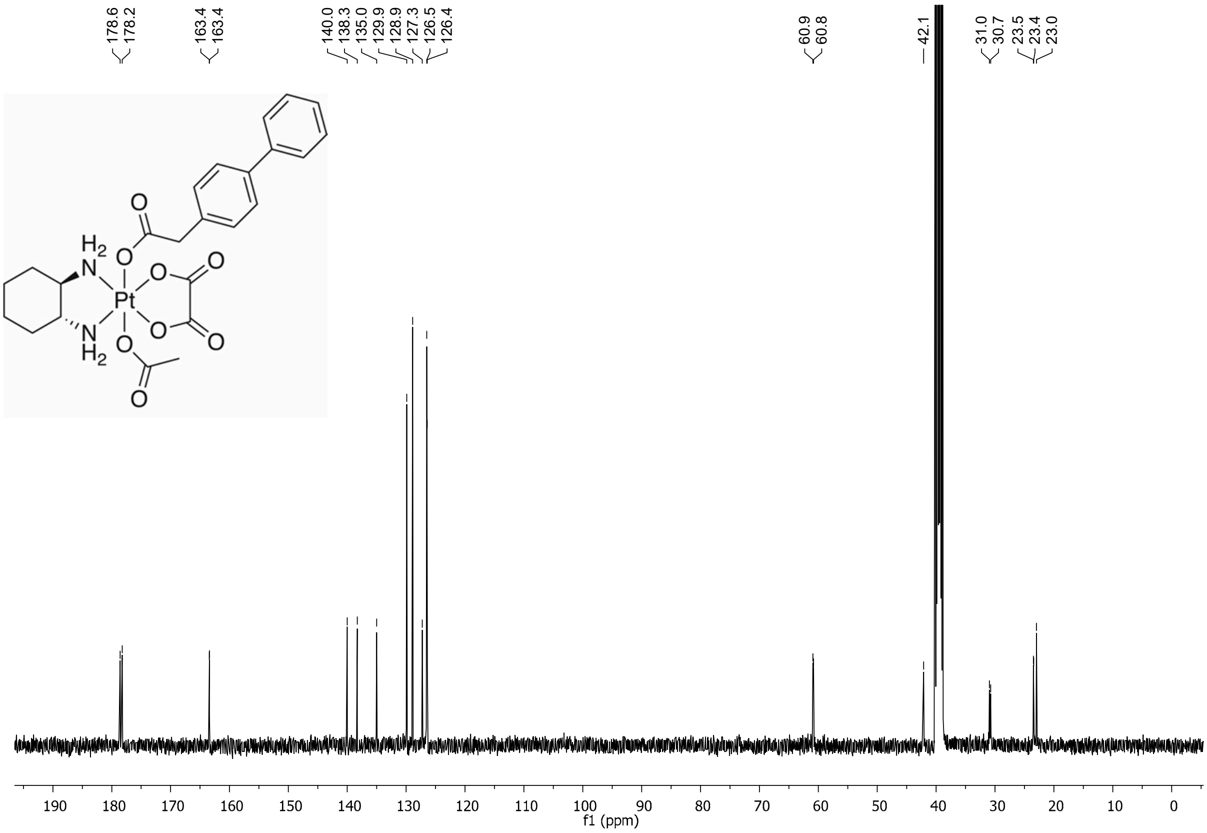

Figure S24. $^{13}C\{^{1}H\}$ NMR spectrum of **12** (DMSO-d$_6$, 101 MHz).

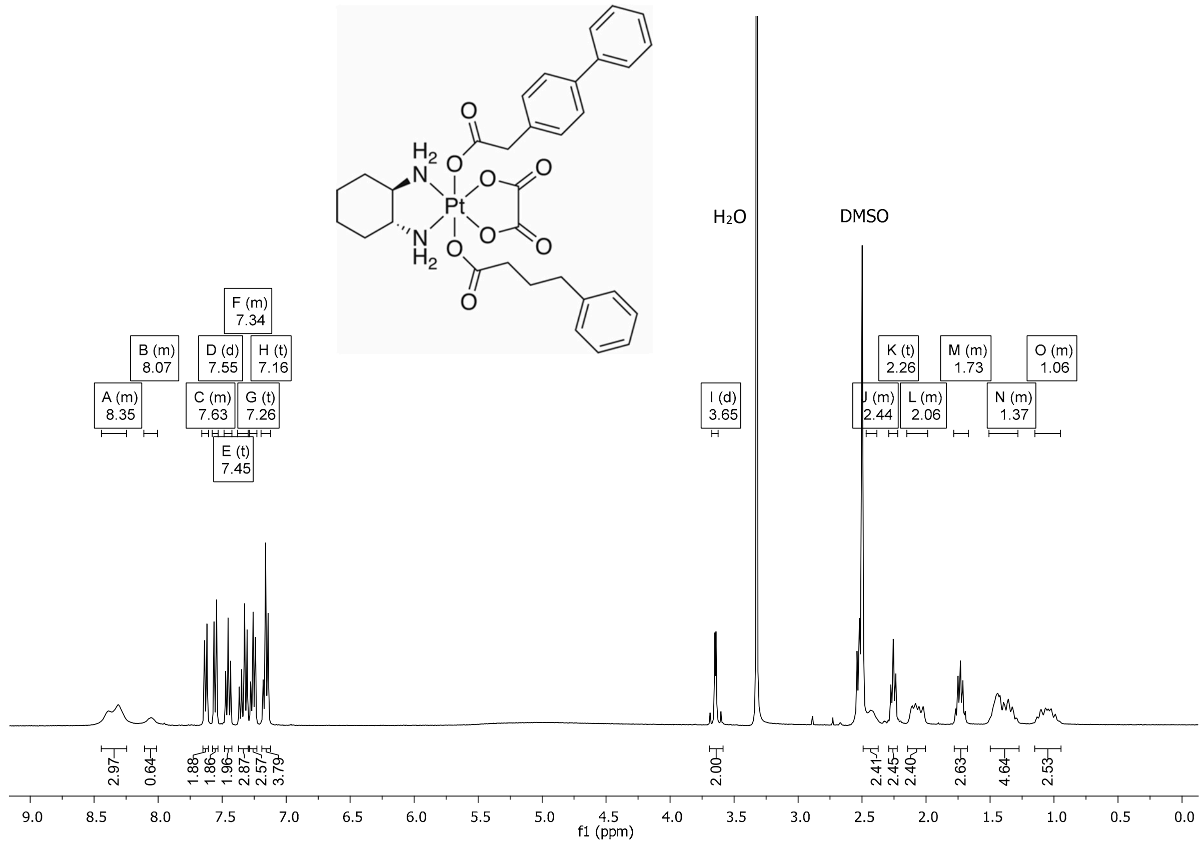

Figure S25. ^{1}H NMR spectrum of **13** (DMSO-d_6, 400 MHz).

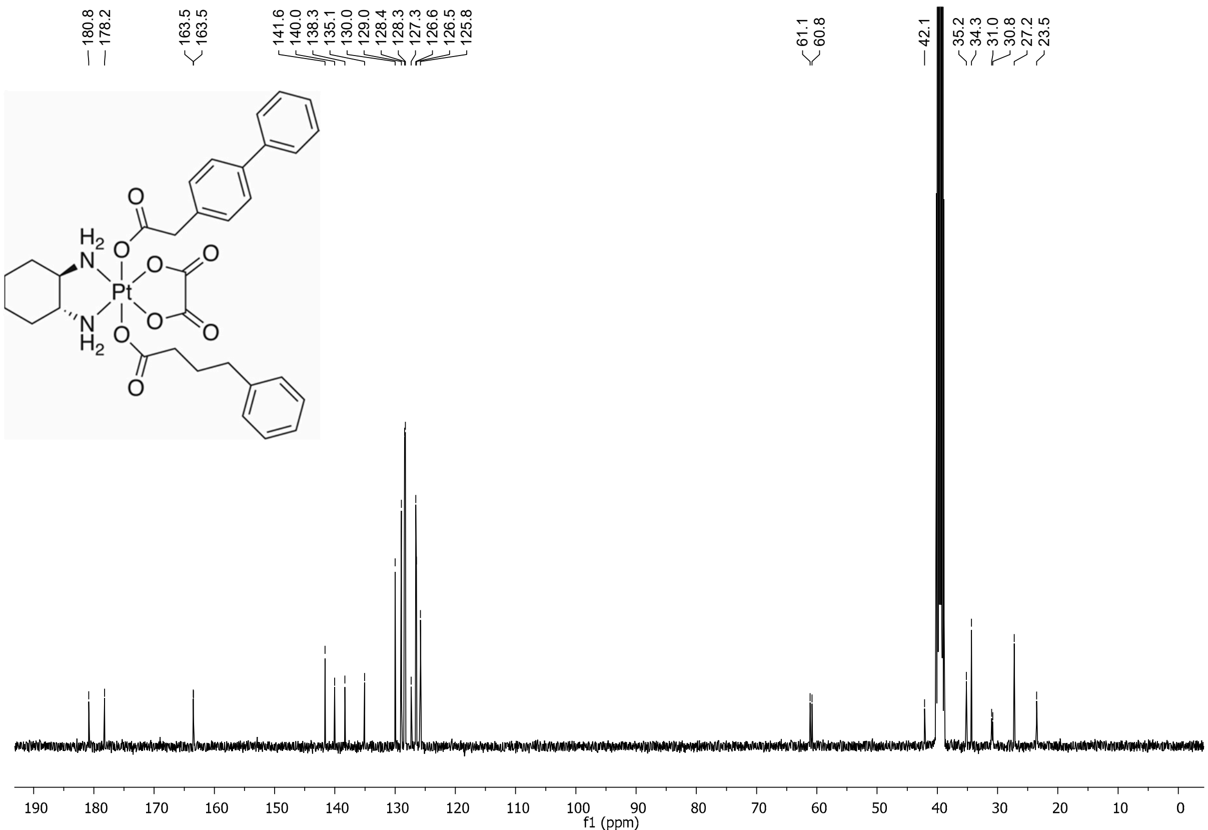

Figure S26. $^{13}C\{^{1}H\}$ NMR spectrum of **13** (DMSO-d_6, 101 MHz).

Stability investigation of complex 7 in different buffer solutions

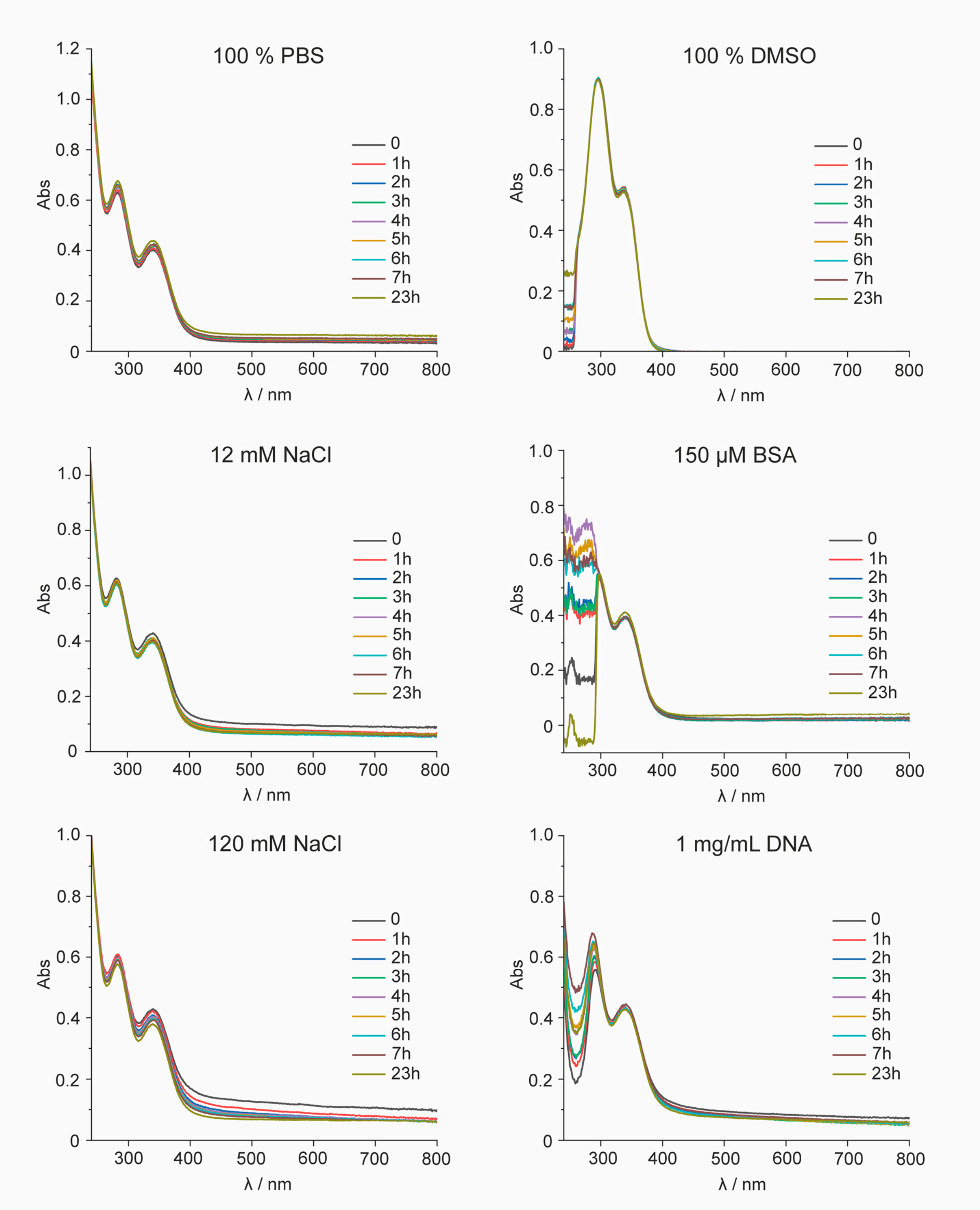

Figure S27. UV-Vis spectroscopic experiment of complex **7** (100 µM) in different buffer solutions over 7 h and after 23 h at 37 °C. Complex **7** was dissolved in DMSO and diluted in the specific solution to receive a concentration of 100 µM with 0.1 % DMSO.

Reduction experiment of complex 7 with ascorbic acid (UHPLC-HRMS)

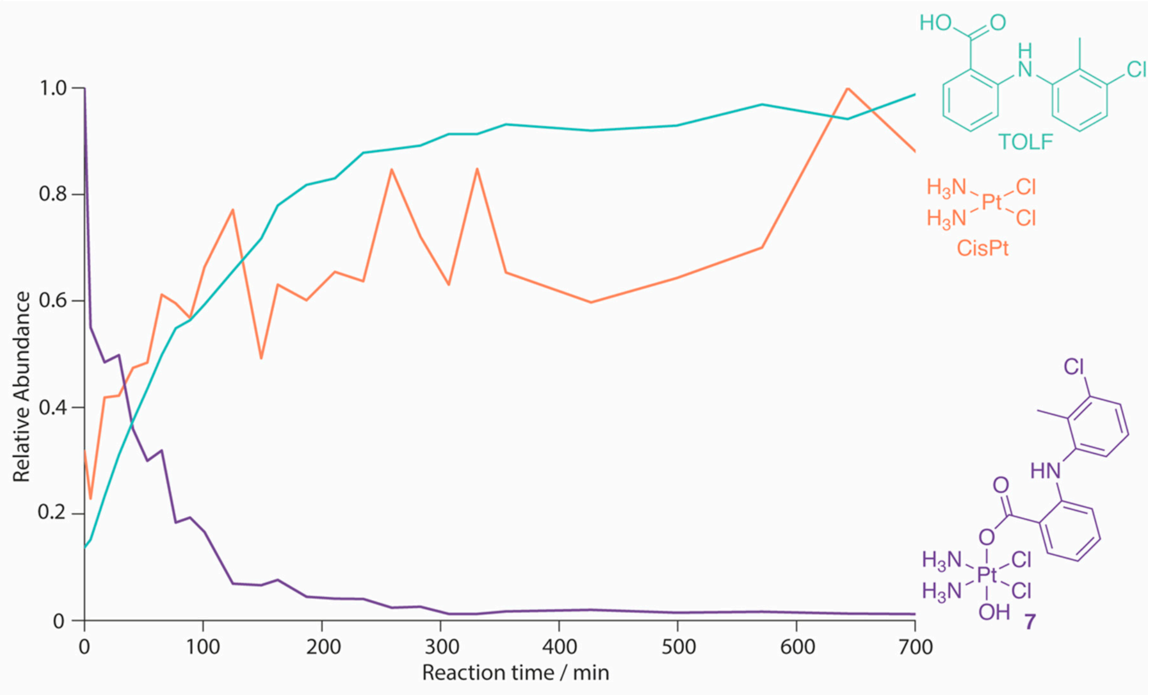

Figure S28. UHPLC-HRMS experiment of complex **7** with ten-fold excess of ascorbic acid in phosphate buffer (pH 7.4) at 37 °C.

COX-1/2 inhibition

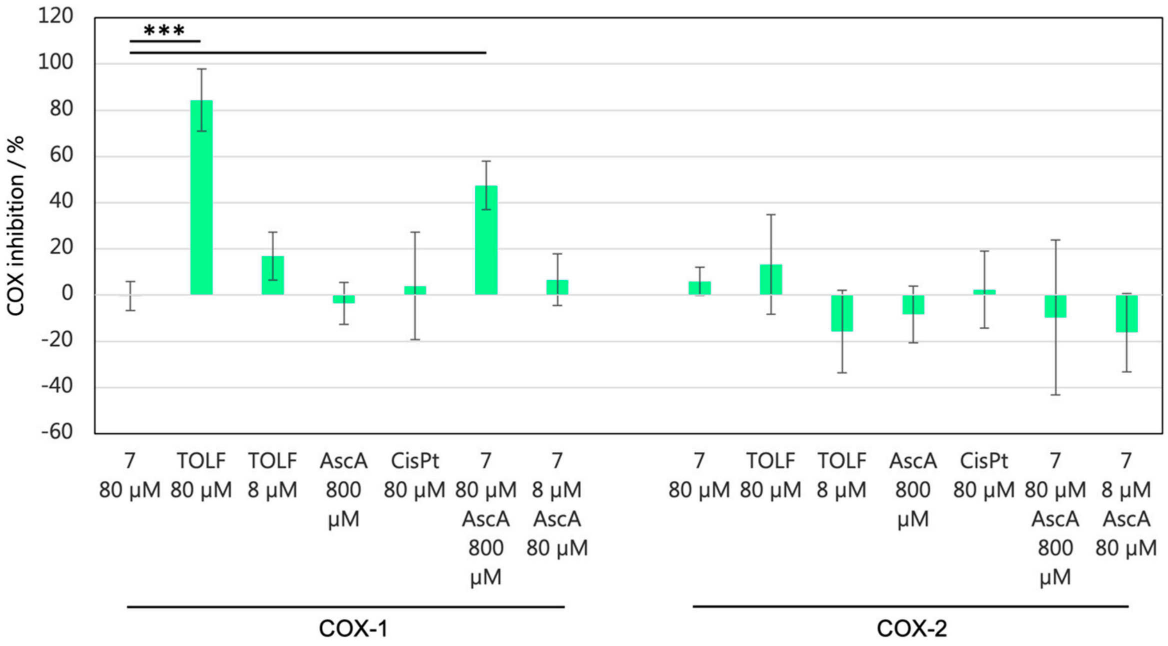

Figure S29. Inhibition of COX-1 or COX-2 activity *in vitro*. Whereas COX-2 could not be inhibited in this assay the free ligand (TOLF, tolfenamic acid) and the reduced complex **#7** (**7** + ascorbic acid, AscA) showed a significant inhibition of COX-1 activity (two-sided student´s t-test, *** $p < 0.005$).

Cell death rate after cisplatin treatment

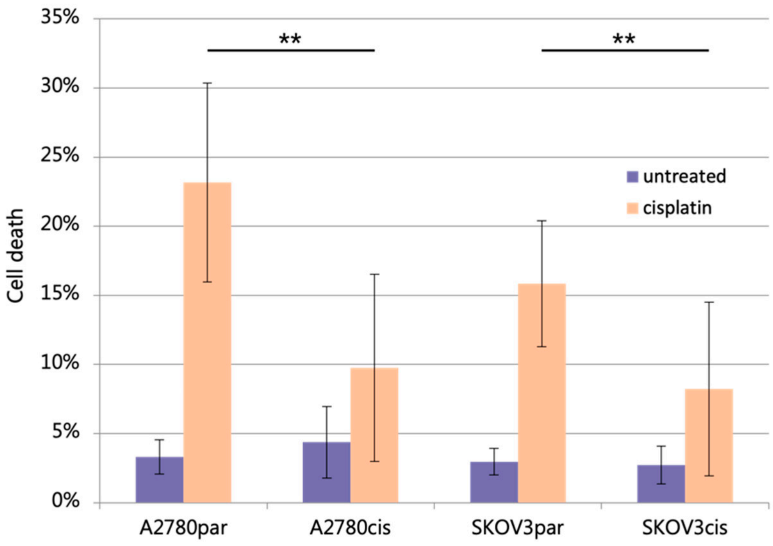

Figure S30. Cell death in % after cisplatin treatment with IC_{50} concentration of parental cells (two-sided student´s t-test, ** $p < 0.01$).

References

1. Ermondi, G.; Caron, G.; Ravera, M.; Gabano, E.; Bianco, S.; Platts, J.A.; Osella, D. Molecular interaction fields vs. quantum-mechanical-based descriptors in the modelling of lipophilicity of platinum(IV) complexes. *Dalton Trans.* **2013**, *42*, 3482-3489.
2. Shi, Y.; Liu, S.A.; Kerwood, D.J.; Goodisman, J.; Dabrowiak, J.C. Pt(IV) complexes as prodrugs for cisplatin. *J. Inorg. Biochem.* **2012**, *107*, 6-14.
3. Tolan, D.A.; Abdel-Monem, Y.K.; El-Nagar, M.A. Anti-tumor platinum (IV) complexes bearing the anti-inflammatory drug naproxen in the axial position. *Appl. Organomet. Chem.* **2019**, *33*, e4763.
